# Beta-adrenergic receptors are expressed across diverse cancers

**DOI:** 10.18632/oncoscience.357

**Published:** 2017-08-23

**Authors:** Steven L. Rains, Clarissa N. Amaya, Brad A. Bryan

**Affiliations:** ^1^ Department of Biomedical Sciences, Texas Tech University Health Sciences Center, El Paso, TX, USA

**Keywords:** beta adrenergic, beta blocker, cancer

## Abstract

Based largely on retrospective analyses and a handful of prospective case reports, pharmacological inhibition of the beta adrenergic receptors using beta blockers has shown clinical anti-cancer efficacy in reproductive cancers, as well as angiosarcoma and multiple myeloma. Because of the potential promise of beta blockers as an adjunct to standard anti-cancer therapy, it is imperative to identify other tumor types expressing beta adrenergic (β-AR) receptors so future preclinical and clinical studies can be directed at the most promising tumor targets. We performed immunohistochemical detection of β1-AR, β2-AR, and β3-AR across 29 of the most common human cancer types (389 tissues total) and 19 matching non-diseased controls (100 tissues total). Our analysis revealed all three β-AR receptors were expressed most strongly in melanoma relative to other cancer types. Other malignancies that revealed relatively higher levels of β-AR receptors were esophagus, pancreas, kidney, and lung cancers. Moreover, particular β-AR receptors exhibited significant overexpression in tumor tissue relative to their matching normal tissue in urogenital/reproductive malignancies including breast, endometrium, ovarian, and urothelial cancer, as well as colon, lung, and thyroid cancer. This study identifies several cancer types expressing the β-AR receptors which should be evaluated in future studies for susceptibility to beta blockade.

## INTRODUCTION

Beta adrenergic (β-AR) receptors are G-protein coupled receptors responsible for mediating vasodilation following stimulation by the catecholamines epinephrine and norepinephrine [1, 2]. When activated, the β-AR receptors induce intracellular signal transduction pathways through triggering cyclic adenosine monophosphate (cAMP) and cAMP-dependent protein kinase A cascades [1, 2]. This leads to endothelial nitric oxide synthase (NOS)-mediated release of nitric oxide to induce vasodilation and synthesis of pro-angiogenic factors such as vascular endothelial growth factor and basic fibroblast growth factor to promote angiogenesis [1, 2].

Three different β-AR receptors have been identified in humans, and are widely expressed in vascular tissues [2–5]. β-AR1 receptors are primarily expressed in cardiac tissue where they perform vital roles in regulating cardiac output, and additional expression has been detected in the urinary bladder and cerebral cortex [6]. β-AR2 receptors are expressed at high density in the lung on bronchial smooth muscles, bronchial epithelial cells, and multiple immune cells [7, 8]. β-AR3 is abundant in brown adipose tissue and performs significant roles in lipolysis and thermogenesis [9].

The β-AR signaling pathway drives the pathogenesis of the benign vascular tumor infantile hemangioma [10, 11] and several malignant tumor types including angiosarcoma [12–16], breast cancer [17], and ovarian cancer [18]. Pharmacological inhibition of the β-AR receptors with beta blockers has shown clinical efficacy against infantile hemangiomas and angiosarcomas [10, 12-15, 19] and based on retrospective studies of large patient cohorts, beta blockers contribute to improving overall survival and decreasing tumor proliferation across a several common cancer types [17, 18, 20–22]. Due to these findings, in late 2016 the beta blocker propranolol was assigned Orphan Drug Designation in Europe for use in soft tissue sarcomas. With promises of a potentially low cost adjuvant therapy for certain cancers, it is imperative to determine if other more common cancers express the β-AR receptors, and thus could be clinically susceptible to beta blockade.

To accomplish this, we determined the expression of β-AR1, β-AR2, and β-AR3 receptors across 29 tumor types representing the most common cancers found in humans and 19 matching normal tissues using high density tissue microarrays. The knowledge gained from this study will allow researchers and clinicians to efficiently target the most promising cancer types that express the β-AR receptors.

## RESULTS

Numerous studies have examined the expression of the β-AR receptors primarily in the heart, lung, and vascular tissues due to the effects of beta blockers on these tissues [23]. Interestingly, new databases such as GTEx Portal (www.gtexportal.org), BioGPS (www.biogps.org), and the Human Protein Atlas (www.proteinatlas.org) reveal that β-AR mRNA and protein are surprisingly expressed at variable levels across a large number of normal and diseased tissues.

**Figure 1:**
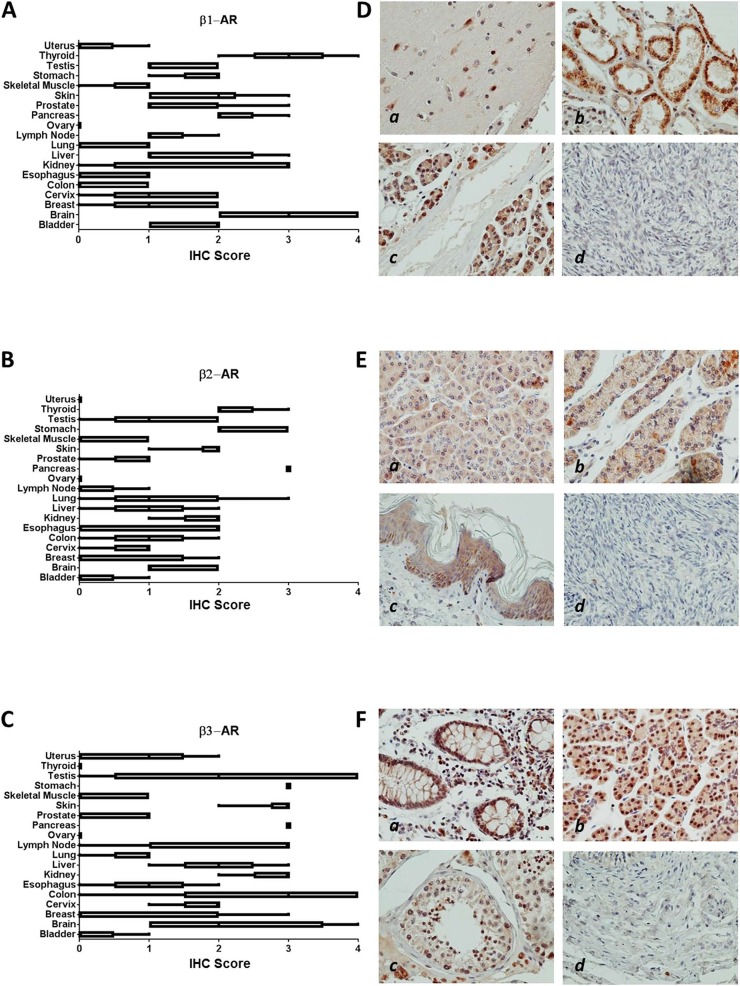
β-AR receptor protein expression in normal tissues **(A-C)** Bar and whiskers plots of IHC staining for β1-AR (A), β2-AR (B), and β3-AR (C) across 19 normal human tissues. The staining intensity was evaluated using the following criteria: 0 = undetectable staining, 1 = equivocal/diffuse staining, 2 = weak staining, 3 = moderate staining, and 4 = strong staining. **(D)** Representative images of relatively higher β1-AR staining in brain (*a*), kidney (*b*), and pancreas (*c*), and no staining in ovary (*d*). **(E)** Representative images of relatively higher β2-AR staining in pancreas (*a*), stomach (*b*), and skin (*c*), and no staining in ovary (*d*). **(F)** Representative images of relatively higher β3-AR staining in colon (*a*), pancreas (*b*), and testis (*c*), and no staining in bladder (*d*).

We analyzed the expression of β-AR proteins across 19 normal tissues comprising epithelial, mesenchymal, and hematopoietic lineages (Figure 1). Relatively higher expression of β1-AR protein was found in brain, pancreas, and thyroid (Figure 1A); β2-AR was highest in pancreas, stomach, and thyroid (Figure 1B); and β3-AR was highest in colon, kidney, lymph nodes, pancreas, skin, and stomach (Figure 1C). Many normal tissues expressed little to no β-AR receptors, including colon, esophagus, lung, ovary, skeletal muscle and uterus for β1-AR; bladder, lymph node, skeletal muscle, uterus, and ovary for β2-AR; and bladder, skeletal muscle, and ovary for β3-AR. Representative images of β-AR staining across selected normal tissues is shown in Figure 1D-1F.

**Figure 2:**
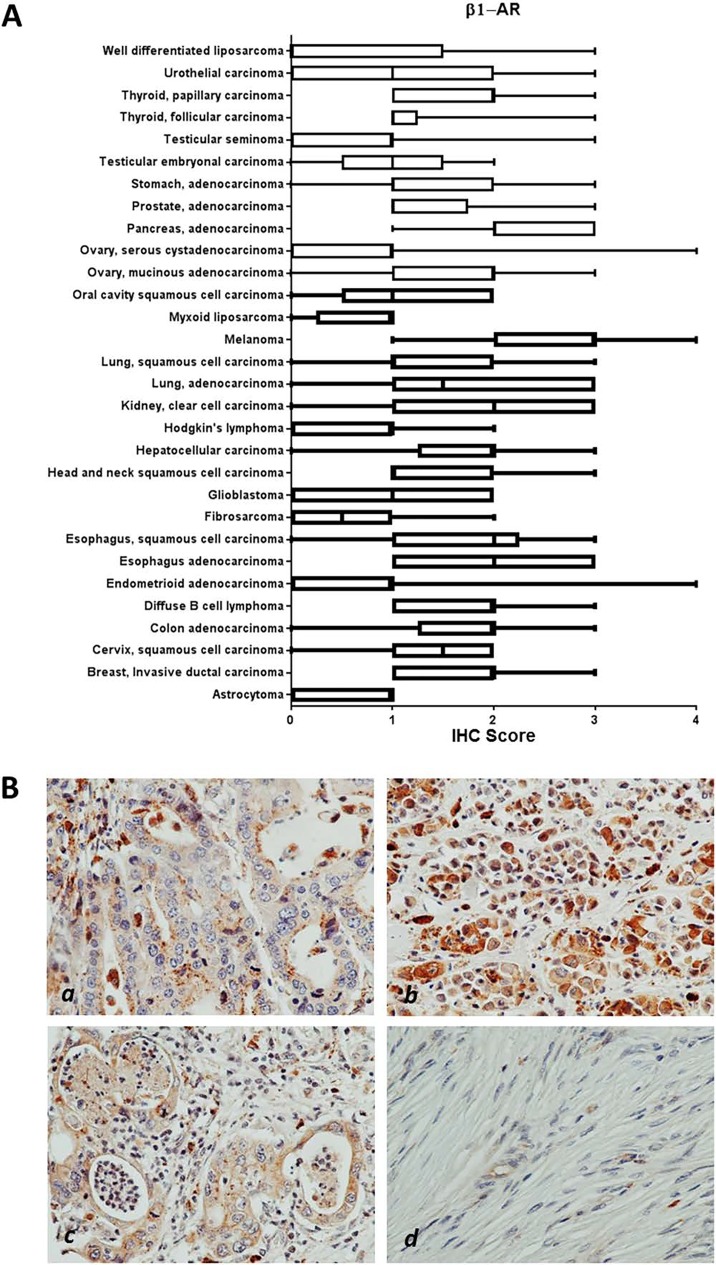
β1-AR receptor protein expression in cancer tissues **(A)** Bar and whiskers plots of IHC staining for β1-AR across 29 human cancer types. The staining intensity was evaluated using the following criteria: 0 = undetectable staining, 1 = equivocal/diffuse staining, 2 = weak staining, 3 = moderate staining, and 4 = strong staining. **(B)** Representative images of β1-AR staining in esophagus adenocarcinoma (*a*), melanoma (*b*), and pancreas adenocarcinoma (*c*), and little/no staining in fibrosarcoma (*d*).

**Figure 3:**
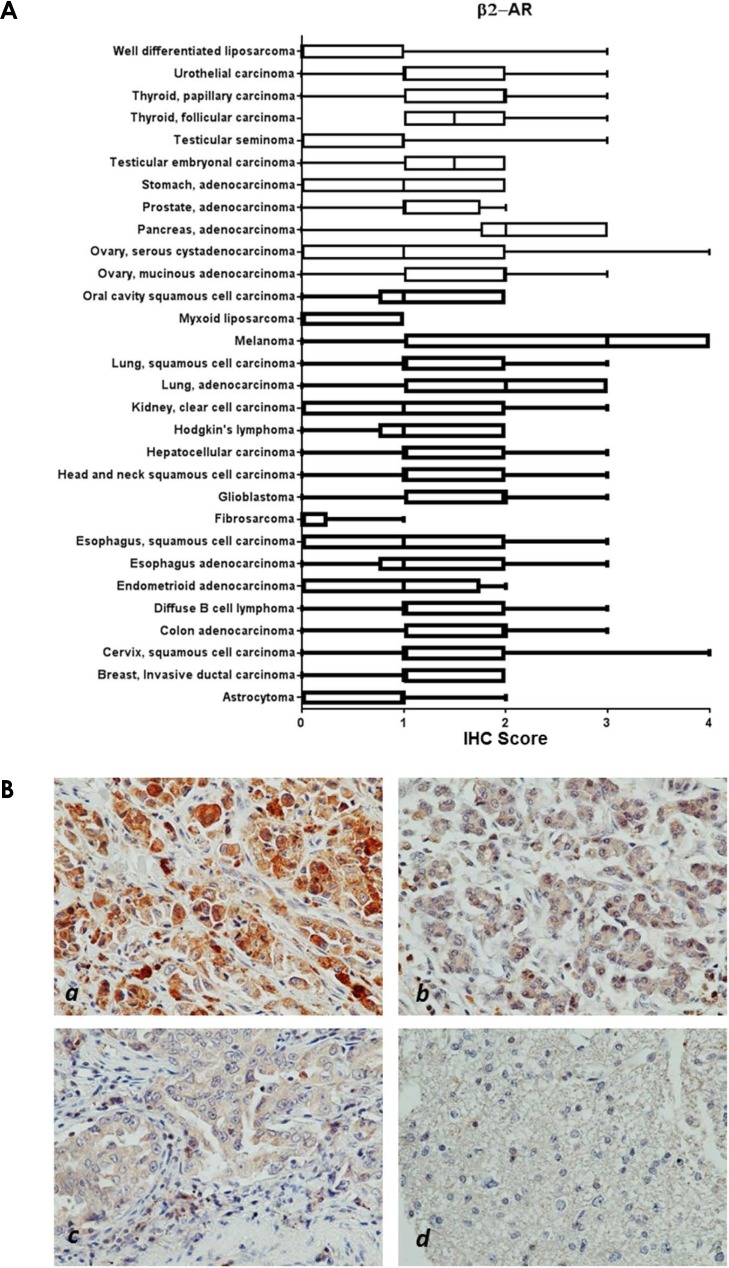
β2-AR receptor protein expression in cancer tissues **(A)** Bar and whiskers plots of IHC staining for β2-AR across 29 human cancer types. The staining intensity was evaluated using the following criteria: 0 = undetectable staining, 1 = equivocal/diffuse staining, 2 = weak staining, 3 = moderate staining, and 4 = strong staining. **(B)** Representative images of β2-AR staining in melanoma (*a*), pancreatic adenocarcinoma (*b*), and lung adenocarcinoma (*c*), and little/no staining in astrocytoma (*d*).

**Figure 4:**
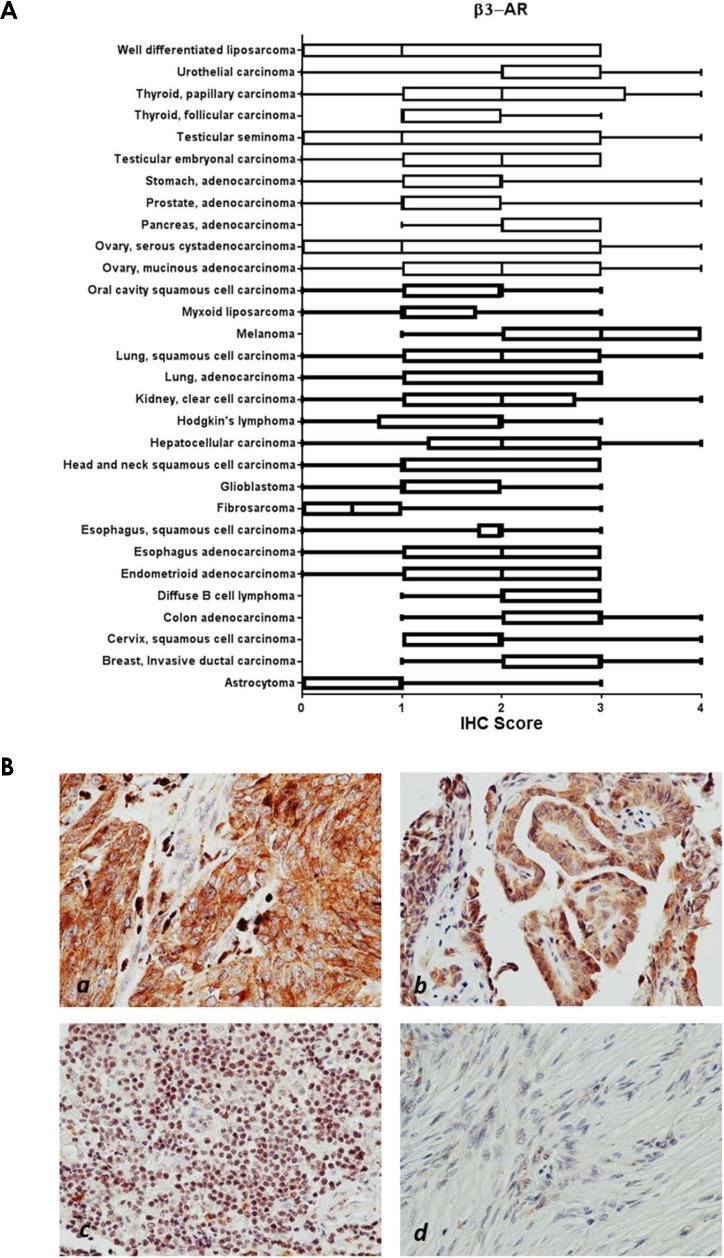
β3-AR receptor protein expression in cancer tissues **(A)** Bar and whiskers plots of IHC staining for β3-AR across 29 human cancer types. The staining intensity was evaluated using the following criteria: 0 = undetectable staining, 1 = equivocal/diffuse staining, 2 = weak staining, 3 = moderate staining, and 4 = strong staining. **(B)** Representative images of β3-AR staining in melanoma (*a*), thyroid papillary carcinoma (*b*), and diffuse B-cell lymphoma (*c*), and little/no staining in fibrosarcoma (*d*).

Within cancer tissues, β1-AR was detected most highly in pancreas adenocarcinoma, melanoma, lung adenocarcinoma, clear cell carcinoma, and esophagus adenocarcinoma (Figure 2). β2-AR was detected most highly in pancreas adenocarcinoma, melanoma, and lung adenocarcinoma (Figure 3). β3-AR was detected most highly in melanoma, and moderate levels of staining were observed across numerous cancer tissues (Figure 4), though the higher levels of β3-AR staining could be attributed to a relatively high affinity of the antibody to its antigen. Across all cancer tissues tested, melanoma exhibited the highest level of β-AR receptor expression.

To evaluate if the β-AR receptors are overexpressed in any of the cancers analyzed in this study, we performed the Mann-Whitney rank sum test to compare IHC scores between normal and cancer tissues. Using a very strict statistical cutoff (p<0.01), we identified a number of cancers that exhibited overexpression for individual β-AR receptors (Table 1). Interestingly, urogenital/reproductive cancers including breast, endometrium, ovarian, and urothelial cancers were overrepresented tumors exhibiting β-AR receptor overexpression.

**Table 1:** Overexpression of β-AR receptors in cancer

Tissue type	IHC score normal vs cancer	p value
β1-AR		
Colon adenocarcinoma	0.4 vs 1.9	0.001
β2-AR		
Endometrioid adenocarcinoma	0.0 vs 1.1	0.01
Ovary mucinous adenocarcinoma	0.0 vs 1.6	<0.001
Urothelial carcinoma	0.2 vs 1.3	0.01
β3-AR		
Breast invasive ductal carcinoma	0.8 vs 3.0	0.005
Lung squamous cell carcinoma	0.8 vs 2.7	0.01
Ovary mucinous adenocarcinoma	0.0 vs 2.4	<0.001
Ovary serous cystadenocarcinoma	0.0 vs 1.9	<0.001
Thyroid follicular carcinoma	0.0 vs 1.9	<0.001
Thyroid papillary carcinoma	0.0 vs 2.7	<0.001
Urothelial cancer	0.2 vs 2.2	<0.001

IHC scores are presented as the median score for each tissue type.

p value<0.01 (CI=95) was considered statistically significant.

## DISCUSSION

In this study we evaluated the protein expression of the three β-AR receptors across a large panel of normal and cancer tissues, showing that these proteins are absent to weakly expressed across many tissues, with moderate to strong expression observed in select tissues. We additionally discovered that the β-AR receptors were overexpressed in several cancer types, particularly in urogenital and reproductive tumors. Although IHC detection of the β-AR receptors is not necessarily indicative of the level of tumor responsiveness to β-blockade, it does help identify β-AR positive cancers that may benefit from this emerging treatment, while excluding cancers from future studies that do not express these receptors.

The β-AR receptors are expressed in normal vessels [2–5] and have more recently been detected in various vascular tumors including hemangiomas, hemangioendotheliomas, and angiosarcomas [13, 16, 24, 25]. Indeed, beta blockade has shown remarkable efficacy in numerous clinical settings against infantile hemangiomas [10, 19], hemangioendothelioma [26–28], and angiosarcoma [12–15]. Due to the success of propranolol against angiosarcoma, this drug has recently received Orphan Drug Designation by the European Medicines Agency for use against soft tissue sarcomas.

In this study we demonstrated that the β-AR receptors are expressed across multiple cancers, thus it is possible that beta blockade could show efficacy against additional tumor types. Indeed, retrospective studies of individuals taking beta blockers have revealed remarkably improved survival outcomes for patients with multiple cancer types [18, 20, 22], decreased cancer recurrence in stage II breast cancer patients [21], and decreased tumor proliferation rates in stage I breast cancer patients [17]. Due to these findings, a number of clinical trials testing the efficacy of propranolol as an anticancer agent are currently ongoing. The benefit of this report lies in identifying cancer types that express the β-AR receptors which are most likely to benefit from beta blockade therapy, and to potentially exclude tumor types which do not express β-AR receptors and likely would show no benefit from beta blockade. Based on our findings, future studies should focus heavily on melanoma, as all three β-AR receptors were most highly expressed in this cancer type. Supporting evidence for use of beta blockers in melanoma comes from several recent studies, whereby propranolol induces cell cycle arrest of melanoma cells [29], promotes anti-tumor immunity in a melanoma tumor model [30], and inhibits B16F10 melanoma xenografts at low doses [31]. Based on moderate levels of β-AR expression, other tumor types of interest for future study would be cancers of the esophagus, pancreas, kidney, and lung.

Overexpression of the β-AR receptors has been reported in breast cancer [17, 32], and our analysis uncovered multiple cancers which exhibited overexpression of β-AR receptors relative to their normal non-diseased tissue counterparts. These included urogenital/reproductive malignancies including breast, endometrium, ovarian, and urothelial cancer, as well as colon, lung, and thyroid cancer. It is possible that overexpression of the β-AR receptors could impart tumor sensitivity to beta blockade, though a previous report in breast cancer has shown no correlation between the levels of β-AR receptor mRNA in the tumor cells and sensitivity to propranolol [17]. Moreover, β-AR receptors are expressed at high levels in multiple endothelial cell types [2–5], yet exhibit clinically effective selectivity against vascular tumors [10, 19]. Future preclinical and clinical studies should focus on clarifying this conundrum, and identifying reliable biomarkers of propranolol susceptibility in tumors.

The observations reported in this study will help to identify β-AR receptor positive tumors types so that future efforts evaluating the anti-cancer efficacy of beta blockade can be directed at the most promising targets. Furthermore, identification of tumor types with equivocal or non-detectable levels of β-AR receptors will guide future studies in avoiding tumors potentially non-responsive to beta blockade.

## MATERIALS AND METHODS

### Tissue microarrays

Immunohistochemical staining (IHC) was performed on a clinically annotated tissue microarray (US Biomax #MC5003b) containing formalin-fixed paraffin-embedded tumor (N=389) and normal (N=100) tissues from human patients. Hematoxylin and eosin staining was used to confirm the classification of all tumors. The clinicopathological features of these tissues are shown in Table 2.

**Table 2:** Clinicopathological features of patient tissue samples

Pathology	Sample #	Median Age	Sex
Normal Tissues			
Bladder	5	33.2	4M; 1F
Brain	5	36.8	4F; 1M
Breast	5	42.8	5F
Cervix	5	48.8	5F
Colon	5	31.8	5M
Esophagus	5	32.4	4M; 1F
Kidney	5	35.8	4M; 1F
Liver	5	38.8	4M; 1F
Lung	5	34.8	2M; 2F
Lymph node	5	32.0	3M; 2F
Ovary	5	36.8	5F
Pancreas	5	33.6	4M; 1F
Prostate	5	34.2	5M
Skin	10	32.6	6M; 4F
Skeletal Muscle	5	37.4	5M
Stomach	5	28.2	4M; 1F
Testis	5	40.6	5M
Thyroid	5	32.6	3M; 2F
Uterus	5	23.6	5F
Cancer Tissues			
Astrocytoma	9	35.2	4M; 6F
Breast, Invasive ductal carcinoma	20	51.1	20F
Cervix, squamous cell carcinoma	20	44.0	20F
Colon adenocarcinoma	20	52.9	12M;8F
Diffuse B cell lymphoma	9	44.7	3M; 6F
Endometrioid adenocarcinoma	20	54.7	20F
Esophagus adenocarcinoma	10	62.6	9M; 1F
Fibrosarcoma	10	35.1	4M; 6F
Glioblastoma	11	48.9	6M; 5F
Head and neck squamous cell carcinoma	15	63.1	12M; 3F
Hepatocellular carcinoma	20	52.0	16M; 4F
Hodgkin's lymphoma	10	30.1	9M; 1F
Kidney, clear cell carcinoma	20	56.1	16M; 4F
Lung, adenocarcinoma	10	56.7	7M; 3F
Lung, squamous cell carcinoma	10	55.6	9M; 1F
Melanoma	20	55.8	14M; 6F
Myxoid liposarcoma	4	53.5	2M; 2F
Oral cavity squamous cell carcinoma	5	49.4	4M; 1F
Ovary, mucinous adenocarcinoma	9	44.0	9F
Ovary, serous cystadenocarcinoma	11	45.0	11F
Pancreas, adenocarcinoma	20	55.3	12M; 8F
Prostate, adenocarcinoma	20	70.5	20M
Stomach adenocarcinoma	20	56.1	17M; 3F
Testicular embryonal carcinoma	5	27.6	5M
Testicular seminoma	15	42.4	15M
Thyroid, follicular carcinoma	10	50.5	1M; 9F
Thyroid, papillary carcinoma	10	41.6	2M; 8F
Urothelial carcinoma	20	59.8	15M; 5F
Well differentiated liposarcoma	6	51.5	4M; 2F

M = male; F = female.

### Immunohistochemistry

Expression of the three β-AR receptors was evaluated using IHC for each tissue section. The primary antibodies used were anti-β1-AR (Abbiotech #250919; 1:100 dilution, citrate induced antigen retrieval), β2-AR (Abbiotech #251604; 1:100 dilution, citrate induced antigen retrieval), or β3-AR (Abbiotech #251434; 1:100 dilution, citrate induced antigen retrieval) under conditions previously reported [16, 17]. The β-AR IHC reactions were visualized using HRP/DAB (ABC) Detection IHC Kit (Abcam #ab64264). The staining intensity specifically in the normal and tumor cells was evaluated using the following criteria: 0 = undetectable staining, 1 = equivocal/diffuse staining, 2 = weak staining, 3 = moderate staining, and 4 = strong staining. The Mann-Whitney rank sum test was used to assess statistical analysis of β-AR expression between the normal and tumor tissues.

## Abbreviations

β-AR: beta adrenergic receptor
β-AR1: beta adrenergic receptor 1
β-AR2: beta adrenergic receptor 2
β-AR3: beta adrenergic receptor 3
cAMP: cyclic adenosine monophosphate
DAB: 3,3′-diaminobenzidine
HRP: horse radish peroxidase
IHC: immunohistochemistry
NOS: nitric oxide synthase
